# Psychosocial Evaluation in Adults With Congenital Heart Disease∗

**DOI:** 10.1016/j.jacadv.2023.100588

**Published:** 2023-09-01

**Authors:** Edward Callus

**Affiliations:** a Department of Biomedical Sciences for Health, University of Milan, Milan, Italy; b Clinical Psychology Service, IRCCS Policlinico San Donato Research and University Hospital, San Donato Milanese, Milan, Italy; c European Congenital Heart Disease Organisation

**Keywords:** adults with congenital heart disease, anxiety, mental health, psychology, psychosocial

**Figure:**
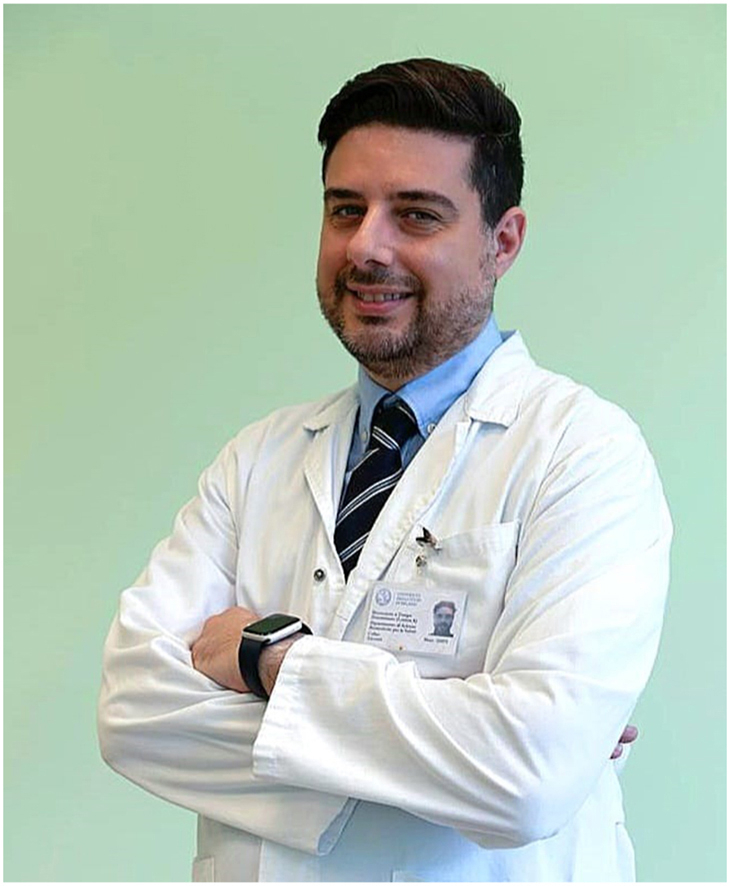

Anxiety is a common and serious psychosocial problem in adults with congenital heart disease (ACHD), affecting their quality of life and health outcomes. However, little is known about the prevalence, predictors, and consequences of preprocedural anxiety in this population, also in comparison to patients with acquired heart disease.^1^^,^^2^ In this issue of *JACC: Advances*, Cook et al^3^ report the results of a multicenter study that assessed the levels and predictors of preprocedural anxiety in patients with ACHD undergoing elective cardiac catheterization, using a validated instrument (the State-Trait Anxiety Inventory) and a control group of patients with acquired heart disease.

Patients with ACHD are a growing and vulnerable population which is more at risk of facing many psychosocial difficulties throughout their lives, when compared to the general population. These include anxiety, depression, post-traumatic stress disorder (PTSD), heart-focused anxiety, coping difficulties, social isolation, stigma, financial stress, and reduced quality of life.^4^ These psychosocial difficulties may be influenced by various factors, such as the complexity and severity of the disease, the number and type of interventions, the transition from pediatric to adult care, the availability and accessibility of specialized care, and the level of social support and education.^5^ Moreover, they may also have adverse effects on the clinical outcomes and health behaviors of patients with ACHD, such as medication adherence, cardiac rehabilitation, and self-management.^6^

One particular circumstance that can trigger or intensify psychosocial challenges in patients with ACHD is the experience of being hospitalized.^7^ It can be associated with heightened levels of anxiety, fear, uncertainty, pain, discomfort, loss of control, and disruption of daily routines. Additionally, it may expose patients with ACHD to negative memories or emotions stemming from previous hospital experiences during childhood or adolescence, potentially leading to PTSD or heart-focused anxiety.^8^ Furthermore, hospitalization can impact the social and occupational functioning of patients with ACHD, affecting their ability to maintain relationships, work, study, or engage in leisure activities.^6^ Consequently, hospitalization can present significant hurdles to the psychological well-being and overall quality of life for patients with ACHD.^4^

The authors found that patients with ACHD had higher levels of trait anxiety, which reflects a stable tendency to experience anxiety in everyday life, but not state anxiety, which reflects a transient response to a specific situation. They also found that state anxiety was strongly associated with trait anxiety, and that trait anxiety was negatively associated with age and positively associated with financial stress. Furthermore, they observed that patients with ACHD with greater disease complexity had higher levels of both state and trait anxiety, although the effect size was relatively small.

This study adds to the growing body of literature that highlights the psychological burden of patients with ACHD^1^^,^^4^ and the need for more attention and intervention from health care providers.^4^^,^^6^ The authors should be commended for conducting a well-designed and executed study that included a diverse sample of patients from 4 tertiary referral centers in the U.S. The use of a validated instrument (the State-Trait Anxiety Inventory) to measure anxiety and the inclusion of a control group of patients with acquired heart disease are important strengths of the study.

The PANIC (Pre-procedural ANxiety In adults with Congenital heart disease) Study sheds light on the elevated levels of trait anxiety in patients with ACHD and emphasizes the importance of addressing anxiety disorders in this population. By recognizing the predictors of anxiety and implementing proactive strategies, health care providers can alleviate preprocedural anxiety and improve the overall well-being of patients with ACHD. Furthermore, the study highlights the significance of conducting research specifically in the preoperative phase, as very few studies have been conducted during this critical period.

The PANIC Study has some limitations that the authors themselves have acknowledged. Its cross-sectional design precludes causal inference regarding anxiety and clinical characteristics or outcomes. Longitudinal studies are needed to examine the temporal relationship between anxiety and disease progression or prognosis in patients with ACHD. The lack of data on clinical outcomes and quality of life limits the evaluation of anxiety's impact on these domains. The voluntary nature of participation may introduce selection bias, potentially limiting the generalizability of the findings. Additionally, the predominantly White study sample from 4 tertiary referral centers in the U.S. may not reflect the diversity or care availability in other settings or regions.

Another limitation of the study is its exclusive focus on anxiety, neglecting other important factors that can affect the well-being and outcomes of ACHD during the preoperative phase, mentioned in the previous paragraphs. Health literacy, which involves obtaining and understanding health information for decision-making and self-management, is crucial for managing the complex nature of ACHD. Social and emotional support, provided by caring individuals during times of need, can help mitigate the negative effects of stress and enhance coping and resilience in patients with ACHD.^4^ Depression, a prevalent mood disorder, can worsen physical symptoms and increase mortality risk in patients with ACHD, yet it is often underdiagnosed or undertreated.^1^ PTSD, stemming from traumatic events like surgeries, can significantly impact patients with ACHD, leading to intrusive memories, avoidance behaviors, and reduced quality of life, yet it frequently goes unrecognized or misdiagnosed.^8^

The findings of this study have several implications for clinical practice. First, they suggest that patients with ACHD may benefit from routine screening and assessment of anxiety, especially during hospitalization and before undergoing invasive procedures. Standardized psychometric screening tools can be used to identify patients at risk of anxiety disorders and refer them to appropriate mental health services. Second, they indicate that patients with ACHD may require more psychological support and education to cope with their medical condition and reduce their preprocedural anxiety. Psychological interventions such as cognitive-behavioral therapy, mindfulness-based stress reduction, or relaxation techniques can help patients manage their negative thoughts, emotions, and behaviors related to their condition and procedure.^4^ Education interventions such as preprocedural information sessions or booklets can help patients understand their diagnosis, treatment options, risks, benefits, and expectations of the procedure. Third, they point to some potential risk factors for anxiety in patients with ACHD, such as younger age, financial stress, and greater disease complexity, which may help identify high-risk subgroups that need more tailored interventions.^9^ For example, younger patients may need more guidance and support in transitioning from pediatric to adult care, coping with developmental challenges such as education, employment, or relationships, and planning for their future health care needs. Patients with financial stress may need more assistance in accessing affordable and adequate health care services or insurance coverage. Patients with complex disease may need more frequent and specialized follow-up care or interventions to prevent or treat complications.

Another important issue that needs to be addressed is the difficulty and availability of receiving mental health care for patients with ACHD. Many ACHD programs do not include psychosocial care, and this is something that should be modified.^4^ Whereas different guidelines outline the necessity of these patients receiving mental health care, in actual fact, in clinical practice it is very difficult that psychologist are available in the programs, mainly for financial reasons. This is a major gap that compromises the quality and continuity of care for patients with ACHD. It is extremely important to provide mental health care by professionals who know the population and have the necessary professionalism to care for them, and ideally, the mental health care professionals should be included in the ACHD program and team, not consultants. Mental health care should be integrated and coordinated with medical care, and should address not only anxiety but also other psychosocial factors that may affect the well-being and outcomes of patients with ACHD.^8^

In case of the absence of mental health care providers in ACHD, the health care providers and/or hospital administrators can reach out to nonprofit associations to assist them with providing mental health care, both during hospitalization and once they are discharged.^10^ Nonprofit associations can offer valuable resources and support, such as peer support groups, educational materials, advocacy activities, or financial assistance. Health care providers can collaborate with these associations to provide additional support and resources to patients with ACHD during hospitalization and beyond. This collaborative approach ensures a comprehensive and holistic approach to addressing the psychosocial needs of patients with ACHD.^10^

In conclusion, the PANIC Study by Cook et al^3^ provides valuable insights into the prevalence and correlates of preprocedural anxiety in patients with ACHD and highlights the importance of addressing the psychological needs of this population. Anxiety is a burden that may affect not only the well-being but also the health outcomes of patients with ACHD. By recognizing the predictors of anxiety, conducting comprehensive psychosocial evaluations, and implementing tailored interventions, health care providers can alleviate preprocedural anxiety and improve the overall well-being and outcomes of patients with ACHD during hospitalization. Further research and collaborative efforts are necessary to optimize mental health care and ensure better outcomes for this unique patient population.

## Funding support and author disclosures

This study was partially supported by Ricerca Corrente funding from the Italian Ministry of Health to IRCCS Policlinico San Donato. Dr Callus has reported that he has no relationships relevant to the contents of this paper to disclose.
